# Does the distribution of health care benefits in Kenya meet the principles of universal coverage?

**DOI:** 10.1186/1471-2458-12-20

**Published:** 2012-01-10

**Authors:** Jane Chuma, Thomas Maina, John Ataguba

**Affiliations:** 1 Kenya Medical Research Institute (KEMRI)-Wellcome Trust Research Programme, P.O Box 230, Kilifi, Kenya; 2 Centre for Tropical Medicine, Nuffield Department of Clinical Medicine, University of Oxford, Headington, Oxford, UK; 3 Ministry of Medical Services, Kenya; 4 Health Economics Unit, Department of Public Health and Family Medicine, University of Cape Town, Rondebosch, 7701 Cape Town, South Africa

## Abstract

**Background:**

The 58^th ^World Health Assembly called for all health systems to move towards universal coverage where everyone has access to key promotive, preventive, curative and rehabilitative health interventions at an affordable cost. Universal coverage involves ensuring that health care benefits are distributed on the basis of need for care and not on ability to pay. The distribution of health care benefits is therefore an important policy question, which health systems should address. The aim of this study is to assess the distribution of health care benefits in the Kenyan health system, compare changes over two time periods and demonstrate the extent to which the distribution meets the principles of universal coverage.

**Methods:**

Two nationally representative cross-sectional households surveys conducted in 2003 and 2007 were the main sources of data. A comprehensive analysis of the entire health system is conducted including the public sector, private-not-for-profit and private-for-profit sectors. Standard benefit incidence analysis techniques were applied and adopted to allow application to private sector services.

**Results:**

The three sectors recorded similar levels of pro-rich distribution in 2003, but in 2007, the private-not-for-profit sector was pro-poor, public sector benefits showed an equal distribution, while the private-for-profit sector remained pro-rich. Larger pro-rich disparities were recorded for inpatient compared to outpatient benefits at the hospital level, but primary health care services were pro-poor. Benefits were distributed on the basis of ability to pay and not on need for care.

**Conclusions:**

The principles of universal coverage require that all should benefit from health care according to need. The Kenyan health sector is clearly inequitable and benefits are not distributed on the basis of need. Deliberate efforts should be directed to restructuring the Kenyan health system to address access barriers and ensure that all Kenyans benefit from health care when they need it.

## Background

Achieving universal coverage currently dominates global health debates. In 2005, the World Health Organization (WHO) resolution called for health systems to move towards universal coverage, where everyone has access to "key promotive, preventive, curative and rehabilitative health interventions for all at an affordable cost, thereby achieving equity in access" [1]. The 2010 World Health Report was also devoted to universal coverage [2]. Universal coverage involves among other things ensuring that health care benefits are distributed on the basis of need for care and not on ability to pay. The fact that the poor have greater need for health care is not disputed [3]; they have worse health indicators and are less inclined to report illnesses because they perceive them to be a normal feature of life [4], or to avoid taking time off income generating activities [5]. Understanding the extent to which health care benefits are distributed on the basis of need for care is thus an important policy question, which health systems should aim to address.

Benefit Incidence Analysis (BIA) allows an assessment of the distribution of health care benefits across socio-economic groups. Initially developed to assess the incidence of government spending [6], the technique has recently been applied to both public and private health sectors to highlight overall health system performance [7]. BIA studies were initially conducted in low-income countries in the 1990s. Findings from these studies revealed that public health care funds hardly reached the poor in most countries [4,8,9]. While these studies were instrumental in highlighting the distribution aspects of various health systems, they have been criticized for using unreliable data sources and applying crude estimation techniques, with limited attention to methods consistency [10]. Moreover, these studies were conducted close to two decades ago, making it important to conduct more rigorous analysis at a time when countries are considering implementing various reforms to support universal coverage. Another limitation of most BIA studies is that they are conducted at one point in time and do not allow for any assessment of distribution changes over different periods. Also, most BIA studies do not assess whether benefits are distributed on the basis of need [7].

This paper addresses some of these challenges by conducting a system-wide analysis of the distribution of health care benefits in Kenya and compares changes in the distribution over two time periods. The paper documents the distribution of health care benefits across socioeconomic groups and assesses the appropriateness of the distribution (i.e. whether benefits are distributed on the basis of need). The study presents the most complete perspective on the distribution of health care benefits in Kenya and plays an important role in highlighting inequities in the Kenyan health system.

### Health care financing and delivery in Kenya

Health services in Kenya are provided by both the public and private sector. The government is the main provider of health services owning 51% of all health facilities [11]. The private for-profit owns 34.3% of total facilities, while the private not-for-profit (largely faith-based institutions) owns 14.8%. Households are the largest source of health funds in Kenya, contributing about 35.9% of total health expenditure [12]. Donor funds account for 31% of total health care funding, while government spending accounts for 29.3% [13]. About 50% of the public health budget is spent at the hospital level [14]. All government health facilities charge user fees for service. In 2004, user fees at dispensaries and health centers were replaced with a flat consultation fee of Kenya shillings 10 (US$0.13) and 20 (US$0.26) respectively.

## Methods

### Data sources

Data were obtained from nationally representative cross-sectional household surveys conducted by the Ministry of Health in 2003 and 2007. The two cross-sectional surveys were designed in a similar way and used the same questionnaire. Key features of the surveys are summarized on Table 1. Data on outpatient visits were collected using a four-week recall period, while hospital admissions were collected for the one year preceding the surveys. Both surveys collected comprehensive data on utilization of health care services, including all visits made to health care providers in the recall period. Data on recurrent health expenditure were sought from public expenditure review reports conducted by the Ministry of Health annually as part of tracking government resources. Expenditure data for the private sector were estimated from the household surveys using information on out-of-pocket payments, mainly because private organizations are not required to submit their expenditure reports to the central level unlike the government facilities, where the same are easily obtained from government reports. It was assumed that private providers will transfer all their expenses to the clients since their goal is profit maximization. To validate this approach, four hospitals (2 private and 2 private-not-for profit) were approached to provide the authors with expenditure data and there were no significant differences with expenditure data estimated through out-of-pocket payments. Ethical approval was obtained from the Kenya Medical Research Institute (Protocol number 1609).

**Table 1:** Key features of the household surveys

	2003	2007
Number of households interviewed:		

• Rural households (%)	5,852 (69.6)	5,810 (68.7)

• Urban households (%)	2,552 (30.4)	2,643 (31.3)

Number of individuals		

• Male (%)	18,765 (49.2)	18,787 (49.2)

• Female (%)	19,225 (50.5)	19,436 (50.9)

Individuals reporting illness in the last four weeks (%)	6,279 (16.5)	5621 (14.7)

Individuals hospitalized in the last 12 months (%)	504 (1.5)	764 (20.)

Individuals seeing preventive care in the last four weeks (%)	2,464 (6.5)	1,644 (4.3)

### Data analysis

Standard BIA studies estimate the value of subsidy received from use of public health services. Benefits are estimated in this study using the approach described by O'Donnell et al. (2008), with some adjustments to allow for analysis of private sector services [7]. Briefly, BIA involves three steps: (1) estimating annual utilization rates in relation to socioeconomic groups; (2) estimating unit costs per health service (both outpatient visits and inpatient days), and multiplying the unit costs by services used to determine the monetary benefit of each service; (3) the distribution of health benefits across socioeconomic groups is examined. Where the focus is on the government subsidy, out-of-pocket-payments paid by the service users are subtracted from total benefits. Table 2 presents the estimated unit costs for different levels of care.

**Table 2:** Unit costs by facility type in Kenya Shillings

	2003		2007	
**Facility type**				

	**Outpatient**	**Inpatient**	**Outpatient**	**Inpatient**

Government facilities				

• PHC facilities	97.5	-	114.1	-

• District and provincial hospitals	n.a*	n.a	366.4	1099.6

• Teaching and referral hospitals	n.a	n.a	963.2	2889.5

• All government hospitals	508.5	1523.0	435.2	1306.1

Private not-for-profit				

• PHC facilities	266.8	-	276.3	-

• Hospitals	413.6	1240.9	463.9	13918

Private for profit				

• Clinics	382.6	-	540.9	-

• Hospitals	568.7	1706	551.5	1670.6

*n.a. means that data were not available to distinguish between government facilities

Households were classified into five socioeconomic groups using per capita expenditure. Outpatient utilization rates were converted into annual utilization by multiplying by thirteen [7,10]. Unit costs were estimated by dividing recurrent expenditure for each type of facility by weighted utilization rates. For hospitals that provided inpatient and outpatient care, outpatient visits were converted into inpatient days by dividing by three. This ratio has been widely applied in the literature [15].

There is no single accepted measure of need for health care, although it is widely recognized that health care need varies across socio-economic groups, age and gender [3]. Mortality and morbidity indicators have been widely used to indicate need, but their application in BIA is limited because most surveys that capture mortality rarely incorporate health care utilization data [7]. Other variables frequently used to indicate need include utilization of health care services and self-reported illnesses, but these approaches have been criticized for failing to capture need among the poorest population who do not use health care services due to financial, geographical and cultural barriers and who tend to underreport illnesses due to differences in perceptions on ill health [5]. To address these limitations, need in this study was measured using Self-Assessed Health Status (SAHS). Individuals in the survey were asked to rate their health compared to others of their age and gender. Four categories of SHAS ranging from very good to poor were provided. Borrowing from Ataguba and McIntyre 2009, individuals were classified into two groups of need: good health (indicating no need for care) if they reported their health status to be very good or good; and poor health (indicating need for care) if they reported their health status to be satisfactory or fair.

## Results

### Self reported illnesses and treatment seeking patterns

Illnesses in the four weeks preceding the survey were reported by 16.5% and 14.7% of individuals in 2003 and 2007 respectively (Table 1). Hospital admissions were reported among 1.5% of individuals in 2003 and 2.0% in 2007. Preventive care was sought by 6.5% of individuals in 2003 and 4.3% in 2007. Table 3 shows the distribution of treatment seeking actions taken in the formal health sector. A total of 4,484 outpatient visits were made to formal health care providers in 2003 compared to 4,736 in 2007. Government health facilities accounted for 57.6% of all outpatient visits in 2003 and 74.3% in 2007. Private clinics were the second largest source of outpatient care, accounting for 21.1% of visits in 2003 and 12.1% in 2007. Inpatient care followed a similar pattern with the majority of hospitalizations occurring in government hospitals (72.4% in 2003; 67.5% in 2007).

**Table 3:** Distribution of health care actions formal care

	2003		2007	
**Actions n (%)**	**Outpatient**	**Inpatient**	**Outpatient**	**Inpatient**

Government PHC	1,418 (31.6)	n.a*	1,726 (37.7)	n.a

Government hospitals	1,169 (26.0)	333 (72.4)	1,672 (36.5)	583 (67.5)

Private-not-for-profit PHC	278 (6.2)	n.a	162 (3.5)	n.a

Private-not-for-profit hospitals	182 (4.1)	58 (12.7)	191 (4.2)	127 (14.7)

Private clinics	947 (21.2)	n.a	554 (12.1)	n.a

Private hospitals	490 (10.9)	68 (14.9)	431 (9.4)	153 (17.7)

Total actions	4,484 (100)	457 (100)	4,763 (100)	863 (100)

*n.a. means facility does not provide inpatient services

### Distribution of outpatient and inpatient benefits by sector

The distribution of health care benefits is presented in Table 4. Public primary health care services (PHC) benefits showed a pro-poor distribution, with the poorest quintile receiving 25.4% share of benefits in 2003 and 25.7% in 2007. The concentration index indicates a greater pro-poor distribution in 2007 (CI = -0.148), compared to 2003 (CI = -0.105). Private not-for-profit PHC benefits showed a pro-rich distribution in 2003 (CI = 0.021) and pro-poor in 2007 (CI = -0.115). Benefits from private clinics show a pro-rich pattern in both time periods, although the pro-rich distribution was greater in 2007 than in 2003 (CI = 0.051 in 2003; 0.102 in 2007).

**Table 4:** Distribution of health care benefits across socioeconomic groups

	Public health facilities			Private-not-for profit facilities			Private-for-profit facilities		
	**PHC**	**Hospital outpatient**	**Hospital inpatient**	**PHC**	**Hospital outpatient**	**Hospital inpatient**	**PHC**	**Hospital outpatient**	**Hospital inpatient**

**2003 survey**									

1 (Poorest)	25.4	13.4	9.5	16.5	15.6	19.7	17.1	11.7	13.0

2	21.6	15.7	18.5	19.0	17.0	9.0	23.0	15.1	13.1

3	18.4	15.5	23.1	19.9	10.0	27.0	15.4	17.8	10.2

4	20.8	26.4	29.4	26.6	26.6	29.7	20.1	27.0	28.5

5 (Richest)	13.8	29.0	19.5	18.0	30.8	14.7	23.9	28.5	35.3

CI	-0.105	0.166	0.119	0.021	0.186	0.025	0.051	0.177	0.279

*p*-value	0.003	0.000	0.04.3	0.699	0.031	0.804	0.262	0.000	0.038

**2007 survey**									

1 (Poorest)	25.7	11.5	14.0	23.4	25.3	30.7	13.4	12.2	11.0

2	25.7	9.9	21.3	18.7	16.8	16.7	19.7	14.3	7.2

3	20.5	20.2	20.8	32.6	20.7	9.8	14.8	15.5	21.4

4	16.2	25.5	20.8	15.3	15.2	29.8	27.9	17.3	14.9

5 (Richest)	12.0	32.9	23.0	10.3	22.0	12.5	24.2	40.3	45.5

CI	-0.148	0.221	0.068	-0.115	-0.055	-0.095	0.102	0.253	0.312

*p*-value	0.000	0.000	0.103	0.144	0.484	0.379	0.025	0.000	0.000

Hospital level out-patient (OP) and inpatient (IP) benefits in all sectors were pro-rich in 2003, but in 2007, private not-for-profit service benefits were pro-poor. The poorest quintile received 13.4% and 9.5% of public hospitals outpatient and inpatient benefits in 2003, while in the same year they received 15.6% and 19.7% of outpatient and inpatient benefits respectively from the private not-for-profit hospitals. Private for-profit sector benefits showed a wider pro-rich distribution with the richest quintile receiving 40.5% and 45.5% of outpatient and inpatient benefits in 2007.

**Figure 1:**
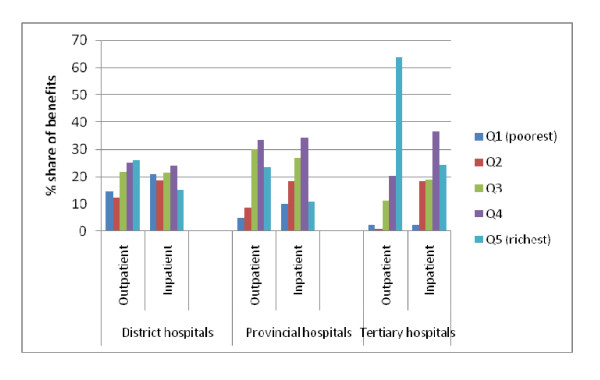
**Distribution of public sector benefits by hospital category (2007)**.

To assess differences within the public health sector, the 2007 results were categorized into different levels of care. Results show a positive relationship between the pro-rich distribution and level of care (Figure 1). The richest quintile received 63.5%, 23.5% and 26.0% share of outpatient benefits for tertiary, provincial and district level facilities respectively. In contrast, the poorest quintile received 2.5%, 4.7% and 14.8% share of tertiary, provincial and district level outpatient benefits respectively.

### Distribution of total health care benefits

**Figure 2:**
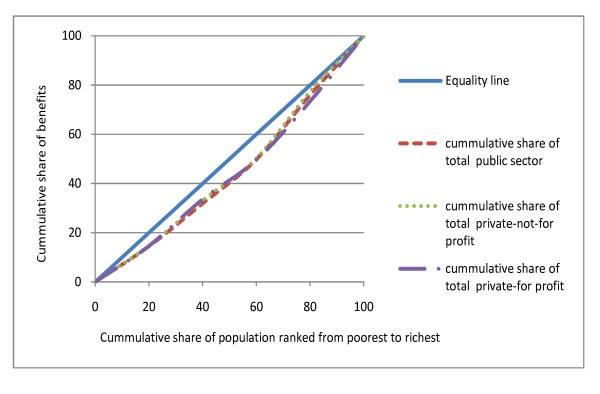
**Distribution of total benefits by sector (2003)**.

**Figure 3:**
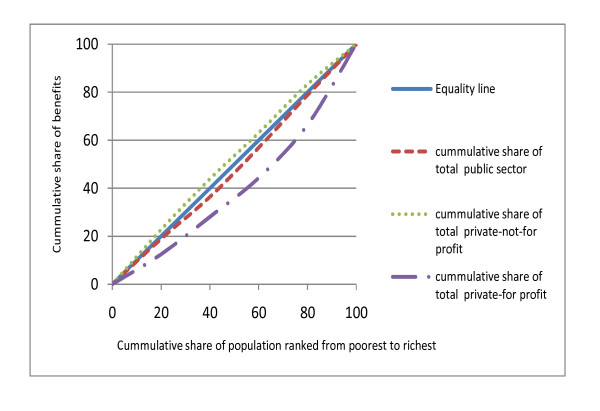
**Distribution of total benefits by sector (2007)**.

When benefits for each sector are combined to arrive at an overall sector distribution, results reveal similar patterns for the public, private-not-for-profit and private-for-profit sectors in 2003; although some differences were noted in 2007 (Figures 2 and 3). Overall, in 2003, about 50% of all hospital level benefits in each sector were received by the richest two quintiles (CI = 0.105 for public facilities; 0.088 for private-not-for-profit; 0.117 for private-for-profit facilities). In 2007, this proportion reduced to 42.1% for public sector benefits and 36.8% for private-not-for-profit benefits, while it increased to 55.6% for private-for-profit benefits. When benefits for each sector are combined to arrive at total health system distribution, the results indicate that the Kenyan health system is generally pro-rich. The richest two quintiles received a 50.2% share of total health systems benefits in 2003 (CI = 0.109) and 46.7% share of total health system benefits in 2007 (CI = 0.077).

### Distribution of government subsidy

Estimating the incidence of public health spending requires that out-of-pocket payments are subtracted from total benefits. Table 5 shows the distribution of the subsidy derived from public health care spending and the mean per capita subsidy across socioeconomic groups. The results reveal a similar pattern as previously observed before out-of-pocket payments were subtracted from health care benefits. Public health care benefits were pro-poor in 2003 (CI = -0.098) and 2007 (CI = -0.17), although differences across socioeconomic status in 2003 were not statistically significant (p = 0.139). Hospital level subsidy was mainly pro-rich for both time periods, except the inpatient subsidy that indicated a pro-poor pattern in 2007 (CI = -0.031). Total government subsidy for all levels of care were pro-rich in 2003 (CI = 0.08; p = 0.035), but differences across socio-economic groups in 2007 were not statistically significant (CI = -0.001; p = 0.960).

**Table 5:** Distribution of government subsidy (percentage share)

	PHC	Hospitals outpatient	Hospitals inpatient	Total subsidy	Mean per capita subsidy in KES	Subsidy as% of consumption expenditure
**2003 survey**						

1 (Poorest)	25.2	16.8	11.2	15.8	125.6	3.0

2	18.7	17.1	14.3	16.4	130.3	1.4

3	21.8	15.1	26.0	19.6	155.9	1.1

4	22.6	28.0	30.9	28.4	225.8	0.9

5 (Richest)	11.6	23.0	17.5	19.8	157.9	0.2

CI	-0.098	0.092	0.120	0.080	0.080	-0.375

*p*-value	0.139	0.000	0.174	0.035	0.032	0.000

**2007 survey**						

1 (Poorest)	26.7	12.8	19.4	19.4	194.9	4.7

2	26.2	11.2	21.7	19.5	195.9	2.2

3	19.7	20.2	20.1	19.8	197.1	1.3

4	16.2	28.5	23.1	23.5	232.6	0.9

5 (Richest)	11.3	27.3	15.7	17.8	170.6	0.2

CI	-0.17	0.176	-0.031	-0.001	-0.001	-0.461

*p*-value	0.000	0.000	0.430	0.960	0.741	0.000

Mean per capita subsidy was higher in 2007 than in 2003 for all socioeconomic groups. In 2003, the poorest quintile received the lowest per capita subsidy of KES 125.6 but in 2007, mean per capita subsidy was lowest among the richest population (KES 170.6). Although the distribution of government subsidy was pro-rich, when the subsidy is expressed as a proportion of household total expenditure, the results indicate a progressive distribution (i.e. the poor received a subsidy that was a larger proportion of their expenditure compared to that received by the richest population).

### Distribution of benefits according to need

**Figure 4:**
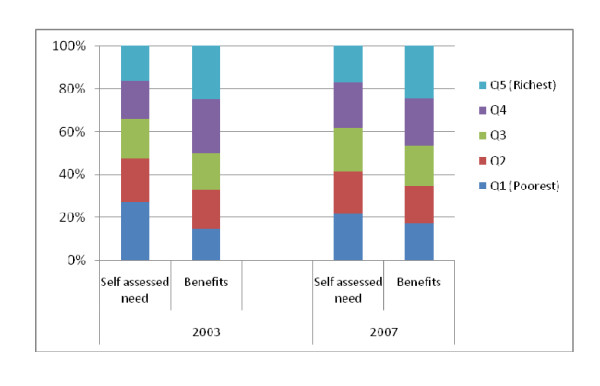
**Distribution of benefits and need for care**.

A limitation of most BIA studies is their failure to assess the extent to which benefits are distributed according to need. Figure 4 compares the distribution of total health system benefits with the need for care (based on SAHS). The results show that the share of self-assessed need differed significantly across socioeconomic groups (p < 0.001 in 2003; 0.08 in 2007). The poorest quintile reported the highest share of need for care in both time periods (27.1% and 21.6% in 2003 and 2007 respectively), but received the lowest share of benefits (14.6% in 2003 and 17.1% in 2003 and 2007). In contrast, the richest quintile received benefits that were significantly higher than their share of need. Differences in the share of benefits received across socio-economic groups were statistically significant (p < 0.001).

## Discussion

### Primary health care facilities are pro-poor

The two poorest quintiles received about half of the public sector PHC benefits in both time periods. There were no significant differences in the distribution of PHC benefits in the private-not-for-profit sector between 2003 and 2007, although the 2007 results suggest a shift towards pro-poor distribution. The greater pro-poor distribution of public PHC benefits reported in 2007 could be attributed to a user fee reduction policy introduced in 2004, where variable user fees in government dispensaries and health centers were abolished and replaced with a flat rate of Kenya Shillings 10 and 20 respectively. Studies in low and middle income countries have shown that the poorest population tends to use PHC more than the rich [4,10]. These results have led for calls to direct additional funds to primary health care services as a way of promoting equity. The Kenyan government regards primary health care facilities as an important part of the health system and various reforms have been implemented to ensure that primary health care facilities offer quality services (including transferring funds directly from the treasury to facilities' bank accounts to minimize delays experienced when they are channeled through the health ministry). While these funds are expected to promote access to PHC services for all, care should be taken to ensure that the poorest populations, who bear the greatest burden of ill-health, continue to benefit from these services.

### Hospital level services are pro-rich, but larger disparities are recorded in outpatient compared to inpatient services

Hospital level services were pro-rich, except the private-not-for-profit sector that showed a pro-poor distribution in 2007. Outpatient services were more pro-rich than inpatient services in the public and private-not-for-profit sectors. These findings differ from others that have reported a more pro-rich distribution for inpatient compared to outpatient services [4,16,17]. The richest quintile received two-thirds and about a quarter of tertiary level outpatient and inpatient benefits respectively, while provincial and district hospitals mainly served middle-income groups. Health care resources in Africa are concentrated at the hospital level [4,14,18]. Kenya spends over 50% of the public health budget on hospitals services [14]; these resources, as clearly demonstrated by the findings, mainly benefit the two richest quintiles. Previous studies have argued that in countries where the private sector is well developed, government subsidies can be better targeted towards the poor, by diverting the demand for health care for the rich population to the private sector [4]. Our findings suggest that this is not easily achieved. Kenya has had a very developed private sector for the last two decades, but public hospital services remain pro-rich. Alternative strategies to ensure that resources allocated to public hospitals benefit everyone who needs them are required.

### The public and private health sector recorded a similar magnitude of inequalities in 2003 but differences across sectors were observed in 2007

The distribution of total benefits in 2003 revealed a similar magnitude of inequalities across the three sectors under consideration. The richest two quintiles received more than half of total benefits in each sector. However, the distribution of health care benefits in 2007 clearly distinguished between the three sectors; the private not-for-profit sector showed a pro-poor distribution, public sector benefits approached equality, while the private for-profit sector was very pro-rich. Few studies have compared the distribution of benefits for the entire health system. The only study that documents distribution of benefits in both public and private sectors reveal wider inequalities in the private sector compared to the public sector [16]. Private health care facilities are perceived to have greater inequities than the public health system [3]. Our findings demonstrate that in some cases, the degree of inequalities can be similar in all sectors. It is not clear why this pattern was observed in 2003, considering that private services often charge higher fees than government facilities. What is clear though is that neither the public nor the private sectors catered for the needs of the poor in 2003, although the 2007 data suggest some improvements in public and private-not-for-profit sectors.

### Government subsidy records a similar distribution

The distribution of government subsidy follows a pattern similar to the benefits, with the poorest population receiving a larger share of PHC subsidy compared to hospital level subsidy. When the subsidy is expressed as a percentage of households' consumption expenditure the poor receive a subsidy that accounts for a higher proportion of expenditure compared to the richest population. Although governments subsidize health services in many countries, evidence suggests that the rich benefit from government subsidies more than the poor. A study conducted in 21 countries revealed that the richest 20% of the population received 26% of total government subsidies, compared to 16% received by the poorest 20% [8]. In a review of the distribution of government subsidy on health, Chu et al. 2000 reported that public health expenditure was well targeted in 21 out of 38 studies and that the poorest 20% received more than the richest 20% when the subsidy is expressed as a percentage of their income or expenditure (i.e. progressive distribution). Sub-Saharan African countries performed poorly for all levels of care. In a country like Kenya, where there is a significant private sector, and where people access public services on the basis of ability to pay, questions regarding the role played by the public health system in addressing inequities remain.

### The distribution of benefits is inappropriate

The poorest population is in greater need of health services than the richest population, and should therefore receive the largest share of health system benefits [3]. Results confirm that the poorest Kenyans have greater health needs, but they receive the least share of total health system benefits. Few studies assess whether the distribution of health system benefits match need for care. A study conducted in the United States reported that the distribution of public spending favored those with higher need for care and increased strongly with health need [19], while in South Africa the distribution of health sector benefits did not match need for care [16]. Countries should work towards restructuring their health systems in a manner that removes the main barriers of access (including but not limited to affordability, availability and acceptability) to ensure that all people can access care when they need it.

### Limitations

This study has several limitations. First, BIA does not account for differences in quality of care. Quality differences exist between facilities. The poorest population might be using lower quality services than the richest population, suggesting that benefits received by the poor would be much lower if quality differences were accounted for. Second, seasonality is an important factor influencing levels of self reported illnesses, treatment seeking behavior and health care costs [20,21]. The analysis did not account for seasonal variations and therefore annual utilization rates may be overestimated (underestimated) depending on the household surveys timings. Thirdly, outpatient days were converted into inpatient day by divided by three. This approach can have implications for cost levels. Fourthly, need in this study is measured through SAHS. This measure does not tell the equidistance between categories, although it has been shown that standardized measures of need compare well with SAHS categories [22]. Finally, multiple measures of need exist, some of which suggest that the rich might have greater need in relation to non-communicable diseases. Future studies should compare appropriateness of health system benefits distribution using different indicators of need.

## Conclusions

The principles of universal coverage require that all should benefit from health care according to need [23]. The Kenyan health sector is clearly inequitable and benefits are not distributed on the basis of need. Deliberate efforts should be directed to restructuring the Kenyan health systems to address access barriers and ensure that all Kenyans benefit from health care when they need it.

## Endnotes

^1^These are small clinics, usually operating in one or two rooms by a single health professional, either a nurse, clinical officer and in few cases medical officers. A few have laboratories operated by a technician who has some 'partnership' with the owner of the clinic.

^2^Data on hospital category were not available for the 2003 survey.

## Competing interests

The authors declare that they have no competing interests.

## Authors' contributions

JC was responsible for the overall design of the study. TM assisted in sourcing the data, cleaning and analysis. JC and JA were involved in the analysis and writing up. All authors read and approved the manuscript.

## Pre-publication history

The pre-publication history for this paper can be accessed here:

http://www.biomedcentral.com/1471-2458/12/20/prepub
